# A case of acquired thrombotic thrombocytopenic purpura induced by acute severe hepatitis E: successfully treated by plasma exchange and rituximab

**DOI:** 10.1186/s12959-023-00507-1

**Published:** 2023-07-10

**Authors:** Yingwei Ou, Yifan Xu, Zhaowang Tan, Lingxiao Pang, Shengqin Li, Qian Li, Wenwei Cai, Yong Nan, Jianfeng Tu

**Affiliations:** 1 Emergency and Critical Care Center, Department of Emergency Medicine, Zhejiang Provincial People’s Hospital (Affiliated People’s Hospital, Hangzhou Medical College), Hangzhou, 310014 Zhejiang PR China; 2 Graduate School of Clinical Medicine, Bengbu Medical College, Bengbu, 230030 Anhui Province PR China

**Keywords:** Thrombotic thrombocytopenic purpura, Hepatitis E virus, Plasma exchange, Rituximab

## Abstract

With its low morbidity and high mortality rates, thrombotic thrombocytopenic purpura (TTP) has imposed a critical physical and economic burden on both society and individuals. Thrombocytopenia commonly occurs in severe liver failure, and a variety of hepatitis viruses are known to induce immune thrombocytopenic purpura. However, TTP is extremely rare in hepatitis E virus infection. We hereby report a case of a 53-year-old male who present with TTP caused by severe hepatitis E, and the patients achieved successful recovery after treatment. Therefore, we propose considering AMAMTS13 testing as an essential and beneficial approach for accurately diagnosing and treating patients with severe hepatitis or infection with notable platelet decline.

**Supplementary Information:**

The online version contains supplementary material available at 10.1186/s12959-023-00507-1.

## Introduction

Thrombotic thrombocytopenic purpura (TTP) is a rare but life-threatening hematological disorder, affecting 1 to 6 individuals per million [1, 2]. In addition to its extremely high mortality rate [3], TTP also has long-term implications on the patients’ quality of life due to exacerbations, relapses, and persistent neurocognitive defects [4]. TTP is characterized by hemolytic anemia, severe thrombocytopenia and organ failure. The pathophysiology of TTP is based on a severe ADAMTS13 deficiency (activity < 10%) [5]. Although the exact cause of TTP remains unclear, previous studies have suggested that autoimmune diseases and other various infections including Human immunodeficiency virus infection (HIV), Hepatitis C (HCV), and Helicobacter pylori, can induce TTP [6]. Moreover, the onset of thrombocytopenia has been associated with acute viral hepatitis [7]. However, TTP caused by hepatitis E is rarely reported. In this report, we present the case of TTP induced by severe hepatitis E in a male patient.

### Case

**Fig. 1:**
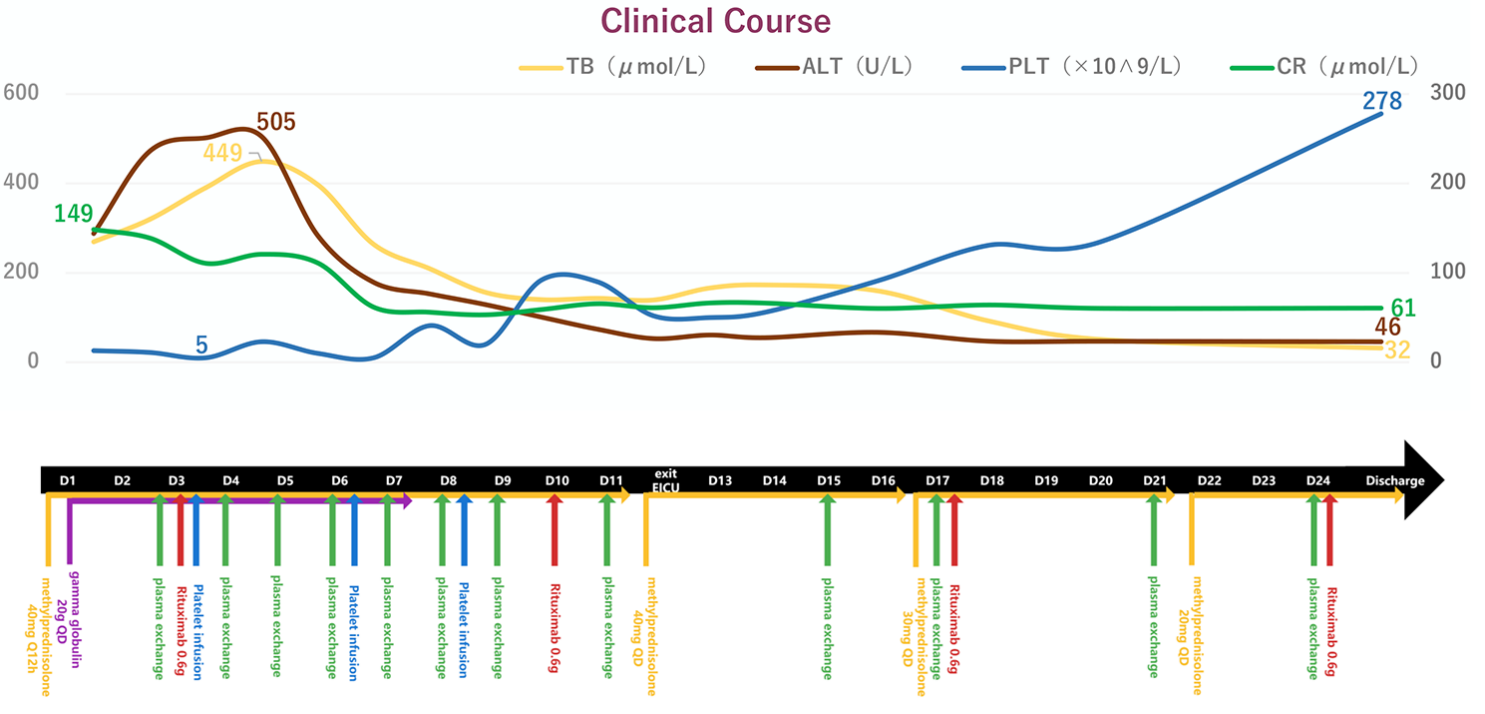
Summary of clinical course during the patient’s hospitalization. Upon admission, the patient received methylprednisolone combined with intravenous immunoglobulin pulse therapy. Methylprednisolone was tapered on day 12, day 17, and day 22, while intravenous immunoglobulin therapy was discontinued after 7 days of treatment. The patient underwent once-weekly treatment with rituximab. Due to limited plasma resources, 12 sessions of intermittent plasma exchange were performed. After receiving three platelet transfusions during the first eight days of hospitalization, the platelet counts finally increased. Platelet recovery occurred spontaneously after clinical improvement, 12 days later. The above curve illustrates the gradual return to normal of total bilirubin, alanine aminotransferase, and creatinine levels following treatment. The patient was transferred from the EICU to a general ward on day 12 and was discharged after recovery

A 53-year-old male was admitted to our hospital due to a persisting fever and jaundice of unknown cause. The patient experienced recurrent fever episodes for 10 days, with the highest temperature reaching 39℃, accompanied by icteric skin and sclera, nausea, fatigue, and myalgia. The patient was a farmer residing in a rural area with good sanitation, and reported no cases of hepatitis among family members. There was no significant medical history. Upon arrival in the emergency room, the patient’s condition deteriorated, with alternating coma and mania accompanied by hypotension. Consequently, the patient was immediately admitted to the emergency intensive care unit (EICU). Upon admission, physical examination revealed a Glasgow Coma Scale score of E3V4M5, and vital signs were as follows: body temperature: 36.1 °C, blood pressure: 110/60 mmHg (while receiving aramine at 1.03 ug/kg/min), pulse: 78/min, respiratory rate: 24/min, oxygen saturation (SpO_2_): 99% (while receiving O_2_ at 3 L/min). However, there were no signs of purpura or skin ecchymosis. The patient’s blood laboratory test results, including inflammatory indicators, liver function, and renal function tests, are presented in Table 1. Based on these results, the patient was diagnosed with acute liver failure (ALF) caused by hepatitis E and thrombocytopenia. However, despite receiving methylprednisolone 40 mg every 12 h combined with gamma globulin 20 g once a day for three days, the patient’s condition did not improve significantly. Furthermore, the patient’s ADAMTS13 activity was 0%, and ADAMTS13 inhibitor was positive. Following the additional diagnosis of TTP, the patient was given plasma exchange of 35ml/kg/day ×12 times, rituximab 0.6 g once a week for 4 consecutive weeks, atomolan 2.4 g once a day for two weeks, transfusion with platelet, meropenem 1 g every eight hours combined with caspofungin 50 mg once a day for anti-infection, anti-shock, and nutrition treatment. Although the patient required intubation due to respiratory failure on the third day of admission, his condition gradually improved following the adjustment to the treatment plan. The ventilator was removed on the 11th day, and the patient was successfully transferred to the general ward for treatment on the 12th day. 26 days after admission, the patient’s condition improved significantly and was discharged. Within the two weeks after discharge, the patient did not experience any relapses. Figure 1 illustrates the patient’s improvement during hospitalization.

**Table 1:** Laboratory findings of the patient during hospitalization

	Day1 (at admission)	Day 3	Day 7	Day 12	Day 25	Two weeks after discharge
WBC (N: 3.5–9.5*10^9/L)	12.37	6.17	15.52	9.99	5.44	6.12
RBC (N: 4.3–5.8 *10^9/L)	3.34	2.51	2.93	2.45	2.29	2.35
PLT (N: 125–350 *10^9/L)	10	23	20	56	254	260
HGB (N: 130–175 g/L)	102	75	89	81	82	113
Hypersensitivity C-reactive protein (N: ＜10 mg/L)	77.4	30.2	5.1	6.9	＜10	/
Procalcitonin (N＜0.5 ng/ml)	2.3	2.0	0.7	0.76	＜0.5	/
TB (N: 3.4–24 umol/L)	319.6	449.2	159.6	173.1	31.7	21.7
DB (N:≤6.8 umol/L)	267.5	376.3	141.4	146.0	14	7.4
ALB (N: 40–55 g/L)	19.4	30.2	30.8	38	36.8	/
CR (N: 57–97 umol/L)	139	120	53.1	66.2	60.0	/
ALT (N: 9–45 U/L)	484	486	129	57	45	53
AST (N: 15–40 U/L)	1283	956	161	55	47	27
GGT (N: 10–60 U/L)	101	54	35	110	92	92
PT (seconds)	16.1	19.4	13.3	12.4	11.2	10.1
Others	IgG-HEV:5.9 (N＜1.0), IgM-HEV:1.3 (N＜1.0) Negative: IgG-HAV, IGM-HAV, IgG-HDV, IGM-HDV, antibody-HCB, IgM-TORCH, syphilis, ANCA, antinuclear antibodies, ammonia	ADAMTS13 activity (%): 0%, inhibitors of ADAMTS13: positive no abnormal cells with bone marrow puncture				

ALB, albumin; ALT, alanine aminotransferase; ANCA, antineutrophilic cytoplasmic antibody; AST, aspartate aminotransferase; CR, creatinine; DB, direct bilirubin; GGT, γ-glutamyl transpeptidase; HGB, hemoglobin; hypersensitivity C-reactive protein; IgG-HAV, immunoglobulin G for hepatitis A virus; IgG-HDV, immunoglobulin G for hepatitis D virus; IgG-HEV, immunoglobulin G for hepatitis E virus; IgM-HAV, immunoglobulin M for hepatitis A virus; IgM-HDV, immunoglobulin M for hepatitis D virus; IgM-HEV, immunoglobulin M for hepatitis E virus; IgM-TORCH, immunoglobulin M for toxoplasmosis, rubella, cytomegalovirus, herpes simplex, and HIV; PLT, platelet count; PT, prothrombin time; RBC, red blood cell; TB, total bilirubin; WBC, white blood cell

## Discussion

Decreased platelet and alterations in platelet function are commonly observed in patients with severe hepatitis [8]. Recent studies suggested that platelets are not only affected by liver disease, but may also contribute to liver disease progression. It is well recognized that thrombocytopenia is caused by decreased platelet production, increased platelet destruction or turnover, and increased platelet consumption in the spleen during virus infection [9]. According to the guidelines, the patient described was immediately diagnosed with ALF caused by hepatitis E virus upon admission [10]. Despite receiving gamma globulin and glucocorticoids for three days, the patient showed no signs of improvement. To obtain an accurate diagnosis, a bone marrow puncture and ADAMTS13 activity test had been performed [11]. A plasma ADAMTS13 activity less than 10 IU/dL (or < 10% of normal) is considered a positive result, suggesting a diagnosis of TTP [12, 13]. The initial management of TTP involves therapeutic plasma exchange and immunosuppressive therapy, including corticosteroids and, increasingly, rituximab [13]. Following the confirmation of TTP diagnosis, we promptly modified the treatment plan, resulting in the patient’s recovery.

Platelets are anucleated blood cells derived from bone marrow megakaryocytes and play a critical role in body hemostasis and thrombosis [14]. In patients with hepatitis E virus-acute liver failure (HEV-ALF), platelets reduction, also known as thrombocytopenia, is an important indicator of poor prognosis [15]. The platelet count can be used to predict 28-day and 90-day mortality in patients with HEV-ALF, with a lower platelet count correlating to a higher risk of death. Considering the important role of platelets in liver disease and the high mortality rate of HEV-ALF [16], an early and accurate diagnosis is crucial before initiating treatment. TTP is a specific type of thrombocytopenia which constitutes a medical emergency, and a delay in implementing appropriate therapy is associated with substantial morbidity and mortality [6, 17].To the best of our knowledge, this is the fourth reported case of TTP associated with hepatitis E, and the first case involving a severely affected male patient who was diagnosed with TTP using an ADAMTS13 test in EICU. All four patients, including a 38-year-old male [18], a 72-year-old male [19] and a 25-year-old male [20], presented with jaundice and no other virus infection except HEV. All previous patients with non-severe liver dysfunction achieved improvement after receiving conventional medical therapy and platelet transfusion. To date, we have not encountered a similar case of HEV-ALF complicated by TTP in the intensive care unit. In fact, it was already recommended that patients with severe TTP should be promptly admitted to the intensive care unit for comprehensive treatment [21, 22]. In the present case, the patient in EICU received close monitoring and comprehensive treatment, including ADAMTS13 testing, assessment of liver function, kidney function, cardiac function and blood clotting indicators. During this period, the patient also required ventilatory support for several days. Following plasma exchange, rituximab administration and platelet infusion, the patient exhibited progressive improvement, and remained in good health two weeks after discharge. The association between hepatitis E infection and severe TTP remains ambiguous, possibly implicating an immune-mediated mechanism and warranting further investigation.

## Conclusion

Patients with severe liver dysfunction should be promptly admitted to the EICU for comprehensive treatment and continuous monitoring of relevant indicators. Additionally, the presented case underscores the importance of ADAMTS13 testing in patients with hepatitis and thrombocytopenia.

## Electronic supplementary material

Below is the link to the electronic supplementary material.

Supplementary Material 1

## Data Availability

The data that support the findings of this study are available from the corresponding author, Jianfeng Tu, upon reasonable request.
