# Supplementary figures and images for: A case of acquired thrombotic thrombocytopenic purpura induced by acute severe hepatitis E: successfully treated by plasma exchange and rituximab
Source: Thromb J. 2023 Jul 10;21:74. doi: 10.1186/s12959-023-00507-1 (PMC10332056; doi:10.1186/s12959-023-00507-1)

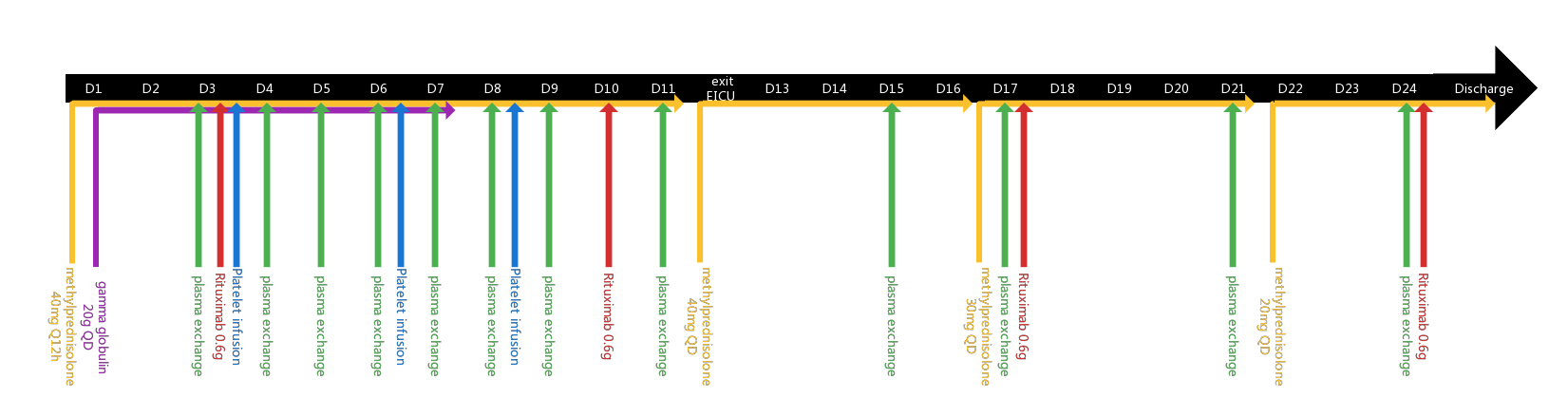

Supplement: Supplementary file 1 — Supplementary Material 1 [file 12959_2023_507_MOESM1_ESM.docx]
